# High-throughput sequencing for the molecular diagnosis of Usher syndrome reveals 42 novel mutations and consolidates *CEP250* as Usher-like disease causative

**DOI:** 10.1038/s41598-018-35085-0

**Published:** 2018-11-20

**Authors:** Carla Fuster-García, Gema García-García, Teresa Jaijo, Neus Fornés, Carmen Ayuso, Miguel Fernández-Burriel, Ana Sánchez-De la Morena, Elena Aller, José M. Millán

**Affiliations:** 1 Grupo de Investigación en Biomedicina Molecular, Celular y Genómica, Instituto de Investigación Sanitaria La Fe (IIS La Fe), Valencia, Spain; 2 CIBER de Enfermedades Raras (CIBERER), Madrid, Spain; 3 Unidad de Genética y Diagnóstico Prenatal, Hospital Universitario y Politécnico La Fe, Valencia, Spain; 4 Servicio de Genética, Fundación Jiménez Díaz, University Hospital, Instituto de Investigación Sanitaria Fundación Jiménez Díaz IIS-FJD, UAM, Madrid, Spain; 5 Unidad de Genética, Hospital de Mérida, Mérida, Badajoz, Spain; 6 Servicio de Oftalmología, Hospital de Mérida, Mérida, Badajoz, Spain

## Abstract

Usher syndrome is a rare disorder causing retinitis pigmentosa, together with sensorineural hearing loss. Due to the phenotypic and genetic heterogeneity of this disease, the best method to screen the causative mutations is by high-throughput sequencing. In this study, we tested a semiconductor chip based sequencing approach with 77 unrelated patients, as a molecular diagnosis routine. In addition, Multiplex Ligation-dependent Probe Amplification and microarray-based Comparative Genomic Hybridization techniques were applied to detect large rearrangements, and minigene assays were performed to confirm the mRNA processing aberrations caused by splice-site mutations. The designed panel included all the USH causative genes (*MYO7A*, *USH1C*, *CDH23*, *PCDH15*, *USH1G*, *CIB2*, *USH2A*, *ADGRV1*, *WHRN* and *CLRN1*) as well as four uncertainly associated genes (*HARS*, *PDZD7*, *CEP250* and *C2orf71*). The outcome showed an overall mutation detection ratio of 82.8% and allowed the identification of 42 novel putatively pathogenic mutations. Furthermore, we detected two novel nonsense mutations in *CEP250* in a patient with a disease mimicking Usher syndrome that associates visual impairment due to cone-rod dystrophy and progressive hearing loss. Therefore, this approach proved reliable results for the molecular diagnosis of the disease and also allowed the consolidation of the *CEP250* gene as disease causative for an Usher-like phenotype.

## Introduction

Usher syndrome (USH) is a rare autosomal recessive disease that associates retinitis pigmentosa (RP), sensorineural hearing loss (SNHL) and, in some cases, vestibular dysfunction. It is the most common form of hereditary disease combining hearing and vision impairment, with a prevalence ranging from 3 to 6.2 per 100,000^1,2^. Three types of USH are distinguished depending on the severity and progression of the pathology: Type 1 (USH I) is typically characterized by a severe-profound congenital hearing loss, onset of RP usually within the first decade of life, and vestibular dysfunction. Type 2 (USH II) patients present with a moderate-severe congenital hearing impairment, a pubertal onset of RP and normal vestibular function. Type 3 (USH III) is defined by progressive hearing loss starting after post-lingual phase and an age-variable onset of RP, whereas the vestibular dysfunction is variable^3^. Despite the three major divisions of the disorder, some patients display a clinical profile not matching any of these categories, being classified as atypical USH.

As well as clinically, USH is genetically heterogeneous. To date, 13 genes have been associated with the disease and these do not explain all the reported cases, suggesting other still unknown genes may be responsible for the disorder^4^.

USH I is commonly caused by mutations in six genes: *MYO7A*, *USH1C*, *CDH23*, *PCDH15*, *USH1G* and *CIB2*. On the other hand, *USH2A*, *ADGRV1* and *WHRN* are the three genes usually responsible for USH II, whilst the *CLRN1* gene is the only one currently associated to USH III cases.

In addition, other genes have been related to the disease. The *PDZD7* gene has been reported to behave as a modifier of retinal disease with *USH2A* and a contributor to digenic inheritance with *ADGRV1*^5^. Recently, *HARS* was proposed as a novel causative gene of USH III, based on a mutation found in two patients^6^ and *CEP250* has been reported as responsible for an atypical Usher syndrome with SNHL and a relatively mild RP^7^.

Most of the USH-causing mutations are private and most of the involved genes are of a large size. These issues can be overcome with the use of high-throughput sequencing (HTS) tools, which enable a rapid, feasible method for the genetic diagnosis of the disease, and they are being increasingly employed^8–12^. The main objective of the present study is the molecular diagnosis of a large cohort of USH patients by means of a HTS screening. Thus, we developed a custom targeted exome design, including the ten disease causative genes and four additional candidates, for its use in Ion Torrent platforms.

## Methods

### Patients

A cohort of 77 USH patients was selected for this study. The probands were classified into the different USH subtypes according to their clinical records. The data (when feasible) consisted of the patient’s ophthalmological studies, including best-corrected visual acuity measurements (BCVA), fundus ophthalmoscopy, visual field examination and electrophysiological examination; and audiological tests^13–15^. Hearing loss severity was established as mild (between >25 and ≤40 dB), moderate (between >40 and ≤70 dB) or severe/profound (>70 dB). Patients presenting a bilateral severe congenital hearing loss (>70 dB), early RP onset and altered vestibular function were diagnosed as USH I. Patients suffering from bilateral congenital moderate-severe hearing loss (40–70 dB) and adolescent-to-adult onset of RP were categorized as USH II. If the patients displayed progressive hearing loss, with or without vestibular dysfunction, and late onset RP, were recognized as USH III. Patients with a profile not quite matching any of these three categories were diagnosed as atypical USH cases. When the clinical data was insufficient, the type was stated as general USH. For case RP1973, further ophthalmological examinations were performed, which included measurements of fundus autofluorescence (FAF), optical coherence tomography (OCT) (acquired with a Heidelberg Spectralis OCT Bluepeak) and visual fields 30-2 and 120-2 strategies by the Humphrey Visual Field Analyzer. Full-field electroretinography was performed according to the International Society for Clinical Electrophysiology of Vision Standards^16^.

From all the patients included, 19 were assigned to a test group in order to evaluate the sequencing platform performance. Eight cases out of these already had a complete molecular diagnosis (at least two USH causative mutations) and 11 where partially solved with only one previously known disease causing mutation. The test group comprised a total of 4 Copy Number Variations (CNVs) and 22 point mutations, represented by variants of different nature (Table 1). Finally, a cohort of 58 previously unscreened USH patients of Spanish origin were recruited for this study in order to determine their genetic diagnosis. Among these to be characterized, 15 were USH I, 31 USH II, and 12 undetermined USH.

**Table 1:** Details of the test group formed by patients carrying previously detected variants in USH genes.

Patient	Phase	Gene	Variant type	Nucleotide	Protein	Class	Reference	Detection
RP692M	*Het*	*USH2A*	Missense	c.14453C > T	p.Pro4818Leu	UV3	Aller *et al*.^54^	Yes
	*Het*	*USH2A*	Nonsense	c.10102C > T	p.Gln3368*	UV4	Jaijo *et al*.^55^	Yes
	*Het*	*USH2A*	Frameshift	c.5278delG	p.Asp1760Metfs*10	UV4	Jaijo *et al*.^55^	Yes
RP1034	*Het*	*CDH23*	Missense	c.8311G > A	p.Gly2771Ser	UV3	Oshima *et al*.^56^	Yes
	*Het*	*PCDH15*	Nonsense	c.733C > T	p.Arg245*	UV4	Ben-Yosef *et al*.^57^	Yes
	*Het*	*PCDH15*	CNV	Deletion exon 3	—	UV4	Aller *et al*.^30^	No
RP1286	*Het*	*PCDH15*	Frameshift	c.1304_1305insC	p.Thr436Tyrfs*12	UV4	Jaijo *et al*.^58^	AFR
RP1495	*Het*	*USH2A*	Frameshift	c.2299delG	p.Glu767Serfs*21	UV4	Liu *et al*.^59^	Yes
RP1522	*Het*	*USH2A*	CNV	Deletion exon 20	—	UV4	Aparisi *et al*.^8^	No
	*Het*	*USH2A*	Frameshift	c.2299delG	p.Glu767Serfs*21	UV4	Liu *et al*.^59^	Yes
RP1537	*Het*	*USH2A*	Missense	c.2276G > T	p.Cys759Phe	UV4	Dreyer *et al*.^60^	Yes
RP1608	*Hom*	*USH2A*	Missense	c.9799T > C	p.Cys3267Arg	UV4	Aller *et al*.^54^	Yes
RP1638	*Het*	*USH2A*	CNV	Deletion exons 5_9	—	UV4	Garcia-Garcia *et al*.^36^	No
	*Het*	*USH2A*	Nonsense	c.5549dupA^a^	p.Tyr1850*	UV4	Garcia-Garcia *et al*.^61^	Yes
RP1639	*Hom*	*USH2A*	Missense	c.10712C > T	p.Thr3571Met	UV3	Aller *et al*.^54^	Yes
RP1740	*Het*	*USH2A*	Frameshift	c.2299delG	p.Glu767Serfs*21	UV4	Liu *et al*.^59^	Yes
RP1746	*Het*	*USH2A*	Missense	c.9799T > C	p.Cys3267Arg	UV4	Aller *et al*.^54^	Yes
RP1757	*Het*	*MYO7A*	In-frame deletion	c.655_660del	p.Ile219_His220del	UV3	Jaijo *et al*.^62^	Yes
RP1768	*Het*	*MYO7A*	Frameshift	c.1623dupC	p.Lys542Glnfs*5	UV4	Bharadwaj *et al*.^63^	AFR
RP1780	*Het*	*PCDH15*	Splice-site	c.3717 + 2dupT	—	UV4	Jaijo *et al*.^58^	Yes
	*Het*	*PCDH15*	Nonsense	c.7C > T	p.Arg3*	UV4	Ahmed *et al*.^64^	Yes
RP1888	*Het*	*USH2A*	Frameshift	c.2299delG	p.Glu767Serfs*21	UV4	Liu *et al*.^59^	Yes
RP1895	*Hom*	*ADGRV1*	CNV	Duplication exons 79_83	—	UV4	Besnard *et al*.^38^	No
RP1906	*Het*	*USH2A*	Frameshift	c.2299delG	p.Glu767Serfs*21	UV4	Liu *et al*.^59^	Yes
RP2019	*Het*	*CDH23*	Missense	c.4488G > C	p.Gln1496His	UV4	Bolz *et al*.^65^	Yes
RP2024	*Het*	*CDH23*	Missense	c.7823G > A	p.Arg2608His	UV3	Astuto *et al*.^66^	Yes

Abbreviations: Het, Heterozygosis; Hom, Homozygosis; AFR, After Filters Relaxation.

^a^This variant was wrongly named in the previous study of reference (Garcia-Garcia *et al*., 2011) as c.5540dupA.

These variants had been previously discovered through other HTS platforms or other variant detection techniques such as MLPA, Sanger sequencing or SNP array.

Segregation analysis was performed by conventional Sanger sequencing when DNA samples of family members were available.

### Ethics Statement

This study was approved by the Hospital La Fe Ethics Committee and authorizations from all the patients and the participating relatives were obtained by signing an informed consent form. All research was performed in accordance with the relevant guidelines and regulations.

### Samples

Genomic DNA (gDNA) from the probands was obtained and purified using standard procedures. The concentration of the resulting DNA samples was determined with Nanodrop and Qubit fluorometer (Thermo Fisher Scientific).

### Targeted USH exome sequencing design

A customized AmpliSeq panel was designed using Ion AmpliSeq Designer tool from Thermo Fisher Scientific (www.ampliseq.com) to generate the targeted library. The designed targeted exome (Table 2) included all exons contemplated in all isoforms of 14 genes: the 10 USH causative genes (*MYO7A*, *USH1C*, *CDH23*, *PCDH15*, *USH1G*, *CIB2*, *USH2A*, *ADGRV1*, *WHRN* and *CLRN1*), the additional locus comprising the c.7595 − 2144A > G intronic mutation in *USH2A*^17^, and 4 USH associated genes (*HARS*, *PDZD7*, *CEP250* and *C2orf71*).

**Table 2:** Details of the target region studied in this study.

Chr	Gene/*locus*	Isoform	Coding exons	Additional exons	Size (bp)	Number of amplicons	Design coverage
11	*MYO7A*	NM_000260.3	48	3	7642	88	98.6%
11	*USH1C*	NM_153676	27	2	3334	38	94.2%
10	*CDH23*	NM_022124.5	69	4	11849	120	99.5%
10	*PCDH15*	NM_033056.3	32	11	8284	67	98.2%
17	*USH1G*	NM_173477.2	3	1	1446	12	100%
15	*CIB2*	NM_006383.2	6	1	684	8	95%
1	*USH2A*	NM_206933	71	1	17043	134	98.9%
1	216064460–216064620^a^	—	—	—	160	1	100%
5	*ADGRV1*	NM_032119.3	89	1	20721	181	99.4%
9	*WHRN*	NM_015404	12	2	2964	26	100%
3	*CLRN1*	NM_174878	3	6	1051	9	100%
5	*HARS*	NM_002109	13	2	1790	14	100%
10	*PDZD7*	NM_001195263.1	17	—	3474	31	97.5%
20	*CEP250*	NM_007186.4	32	—	7969	58	100%
2	*C2orf71*	NM_001029883.2	2	—	3907	23	99.6%

Chr, Chromosome number.

^a^Region of the *USH2A* PE (Pseudo-exon 40) where mutation c.7595 − 2144A > G is located.

The design included a padding of 10 bp of the flanking intronic regions. All the target regions were covered by 810 amplicons, computing a total panel size of 147.95 kb.

### Sequence enrichment and HTS

The amplification of the targets was performed according to the Ion AmpliSeq Library Kit 2.0 protocol (Thermo Fisher Scientific) for Ion Torrent sequencing. The sequencing was carried out with a theoretical minimum coverage of 500x either on the PGM (Ion 318 chip, 500 flows) or Proton system (Ion PI chip, 520 flows).

### Variant filtering and analysis

The resulting sequencing data were analyzed with Ion Reporter Software tool (https://ionreporter.thermofisher.com) in regard to the human assembly GRCh37/hg19. The annotated variants were filtered according to a Minor Allele Frequency (MAF) value ≤0.01, the frequency of the variants was explored in the Exome Aggregation Consortium (ExAC) database, their annotation in the dbSNP (www.ncbi.nlm.nih.gov/SNP/), their description in the Usher syndrome mutation database (https://grenada.lumc.nl/LOVD2/Usher_montpellier/) and the mutation type.

In order to determine the pathogenicity of novel missense or splice-site mutations, the variants were analyzed using several *in silico* prediction tools according to the nature of the mutation. Aminoacid change effects were examinated using the *SIFT*^18^, *PolyPhen-2*^19^ and *PROVEAN*^20^ programs and the additional tools *ATGpr*^21^, *NetStart*^22^ and *TIS Miner*^23^ were applied when concerning translation start loss variants. Putative variants affecting the splicing pattern were investigated with the *Human Splicing Finder 3*.*1*^24^, *MaxEnt*^25^ and *NNSplice*^26^ algorithms.

All the putative pathogenic variants were validated through conventional Sanger sequencing. All the poorly or null covered regions were screened by conventional Sanger for all the cases with only one or none putative disease causing mutations detected through HTS. Additionally, the same patients were screened by Sanger sequencing for recently identified deep intronic mutations that were published after the start of this study and could therefore not be included in the panel design: four in *USH2A*, namely c.14134 − 3169A > G^27^, c.5573 − 843A > G, c.8845 + 628C > T, c.9959 − 4159A > G^28^; and variant c.254–649T > G of *CLRN1*^29^.

### Copy Number Variation analysis

The screening for large rearrangements was performed in all patients where either none or only one mutation was detected with the panel, using either multiplex ligation-dependent probe amplification (MLPA) or a custom microarray-based Comparative Genomic Hybridisation (aCGH).

The MLPA technique (MRC-Holland) allows the identification of large rearrangements for the *USH2A* (probemixes P361 and P362) and *PCDH15* (probemix P292) genes.

In order to screen possible CNVs in the remaining genes, aCGH was designed covering all the genes included in this study. The resulting custom 60 K microarray (Agilent Technologies, AMADID-082310) contained 62976 probes. Three DNA samples with known CNVs from the test group (RP1895, RP1522, RP1034) were used as positive controls in the first batch of the analysis, in order to validate the custom design of the array.The gDNAs were prepared according to the manufacturer’s protocol, as described before^30^.

### Splicing Effect Analysis by Minigenes

Minigene assay was performed for all novel intronic mutations found in this study in order to confirm the splicing alterations, adopting a procedure previously described^31^ and using HEK293 cells. All experiments were performed in duplicate.

### Extended exome screening

Whole exome sequencing (WES) using SureSelect Human All Exon V6 kit (Agilent Techologies) for Illumina platform was performed for sample RP1973 to discard mutations in other genes, in view of the results obtained for this case.

## Results

### Test group

The sequencing results allowed us to identify 20 of the 22 point mutations from the positive controls, while none of the CNVs was detected. When forcing a general parameters relaxation, the two previously undetected changes consisting of frameshift duplications were then recognized. These results and detailed information are summarized in Table 1.

In this study, the second disease causing mutation was detected in 7 out of the 11 patients included in the test group in whom only one of the pathological alleles was previously registered and, therefore, their genetic diagnosis was fulfilled (Table 3). These included 6 novel mutations: two nonsense mutations, two CNVs, 1 frameshift and 1 splice-site mutation.

**Table 3:** Causative mutations and putative pathogenic variants identified in this study

Patient	Type	*Phase*	*Gene*	Variant type	Nucleotide	Protein	Reference or Class
**Patients with two pathogenic mutations**							
RP580M	USH II	***Het***	***ADGRV1***	**Frameshift**	**c.5944dupT**	p.Ser1982Phefs*2	**Novel UV4**
		*Het*	*ADGRV1*	CNV	Dup. exons 79–83	—	Besnard *et al*.^38^
RP689	USH I	*Hom*	*MYO7A*	Missense	c.1190C > A	p.Ala397Asp	Adato *et al*.^67^
RP905	USH II	*Hom*	*USH2A*	Frameshift	c.12093delC	p.Tyr4031*	Garcia-Garcia *et al*.^61^
RP956	USH II	*Hom*	*ADGRV1*	Missense	c.17933A > G^s^	p.His5978Arg	Besnard *et al*.^38^
RP957	USH I	*Hom*	*CDH23*	Missense	c. 6049G > A	p.Gly2017Ser	Roux *et al*.^68^
RP971	USH II	*Het*	*USH2A*	Nonsense	c.12729G > A	p.Trp4243*	Neveling *et al*.^69^
		*Het*	*USH2A*	Missense	c.1531G > A	p.Glu511Lys	Baux *et al*.^70^
RP1350	USH II	***Het***	***ADGRV1***	**Nonsense**	**c.16886G > A**	p.Trp5629*	**Novel UV4**
		***Het***	***ADGRV1***	**Missense**	**c.4102A > T**	**p.Asn1368Tyr**	**Novel UV4**
RP1353	USH II	***Hom***	***ADGRV1***	**Splice-site**	**c.5314 − 5T > A**	p.Asn1772*	**Novel UV4**
RP1399	USH II	***Hom***	***USH2A***	**Nonsense**	**c.11404G > T**	p.Glu3802*	**Novel UV4**
RP1420	USH I	*Hom*	*CDH23*	Nonsense	c.7221C > A	p.Tyr2407*	Besnard *et al*.^50^
RP1495^t^	USH II	*Het*	*USH2A*	Frameshift	c.2299delG	p.Glu767Serfs*21	Liu *et al*.^59^
		***Het***	***USH2A***	**CNV**	**Del. exons 22–49**	**—**	**Novel UV4**
RP1506B	USH II	***Het***	***USH2A***	**Nonsense**	**c.10008C > A**	p.Cys3336*	**Novel UV4**
		***Het***	***USH2A***	**Nonsense**	**c.5416A > T**	p.Lys1806*	**Novel UV4**
RP1564	USH II	***Hom***	***ADGRV1***	**Missense**	**c.14159C > T** ^**s**^	**p.Pro4720Leu**	**Novel UV4**
RP1565	USH II	***Hom***	***USH2A***	**Nonsense**	**c.11404G > T**	p.Glu3802*	**Novel UV4**
RP1567	USH II	*Het*	*MYO7A*	Missense	c.5516T > C	p.Leu1839Pro	Aparisi *et al*.^8^
		***Het***	***MYO7A***	**Start loss**	**c.3G > A**	**p.Met1?**	**Novel UV4**
RP1580	USH I	*Hom*	*MYO7A*	Nonsense	c.6070C > T	p.Arg2024*	Jacobson *et al*.^71^
RP1686	USH II	***Hom***	***ADGRV1***	**Nonsense**	**c.18054G > A**	p.Trp6018*	**Novel UV4**
RP1694	USH I	***Hom***	***USH1G***	**Missense**	**c.1196T > C**	**p.Leu399Pro**	**Novel UV3**
RP1746^t^	USH II	*Het*	*USH2A*	Missense	c.9799T > C	p.Cys3267Arg	Aller *et al*.^54^
		*Het*	*USH2A*	**Splice-site**	**c.12295 − 1G > A**	p.Thr4099Vfs*2	**Novel UV4**
RP1748	USH I	*Hom*	*USH2A*	**Nonsense**	**c.2950C > T**	p.Gln984*	**Novel UV4**
RP1757^t^	Atypical	*Het*	*MYO7A*	IF deletion	c.655_660del^s^	p.Ile219_His220del	Jaijo *et al*.^62^
		***Het***	***MYO7A***	Missense	c.4489G > C^s^	p.Gly1497Arg	Bonnet *et al*.^51^
RP1768^t^	USH I	*Het*	*MYO7A*	Frameshift	c.1623dupC	p.Lys542Glnfs*5	Bharadwaj *et al*.^63^
		*Het*	*MYO7A*	**Nonsense**	**c.6232A > T**	p.Lys2078*	**Novel UV4**
RP1806	USH I	*Hom*	*USH1G*	Nonsense	c.805C > T^s^	p.Arg269*	Aparisi *et al*.^8^
RP1809	USH II	*Het*	*USH2A*	Pseudo-exon	c.7595 − 2144A > G^s^	p.Lys2532Thrfs*56	Vaché *et al*.^17^
		*Het*	*USH2A*	Missense	c.12695C > T^s^	p.Pro4232Leu	Bonnet *et al*.^51^
RP1811	USH II	***Hom***	***USH2A***	**Nonsense**	**c.7932G > A**	p.Trp2644*	**Novel UV4**
RP1857	USH I	***Het***	***CDH23***	**Missense**	**c.3115G > A**	**p.Val1039Met**	**Novel UV3**
		***Het***	***CDH23***	**Missense**	**c.3007T > C**	**p.Ser1003Pro**	**Novel UV4**
RP1869	USH II	***Hom***	***USH2A***	**Missense**	**c.4385C > T**	**p.Thr1462Ile**	**Novel UV4**
RP1872	USH II	***Hom***	***USH2A***	**Splice-site**	**c.5776 + 1G > A** ^**s**^	**p.Gly1858_Thr1925del**	**Novel UV4**
RP1888^t^	USH II	*Het*	*USH2A*	Frameshift	c.2299delG	p.Glu767Serfs*21	Liu *et al*.^59^
		*Het*	*USH2A*	**CNV**	**Dup. exons 28–30**	**—**	**Novel UV4**
RP1900	USH	*Het*	*MYO7A*	**Frameshift**	**c.1934_1935insCCAT**	p.Met645Ilefs*67	**Novel UV4**
		*Het*	*MYO7A*	**Splice-site**	**c.1691 − 1G > A**	p.Phe565Argfs*11	**Novel UV4**
RP1906^t^	USH II	*Het*	*USH2A*	Frameshift	c.2299delG	p.Glu767Serfs*21	Liu *et al*.^59^
		***Het***	***USH2A***	**Nonsense**	**c.9119G > A**	p.Trp3040*	**Novel UV4**
RP1944	USH I	*Hom*	*MYO7A*	Missense	c.3503G > A	p.Arg1168Gln	Aparisi *et al*.^8^
RP1967	USH I	*Het*	*MYO7A*	Nonsense	c.5392C > T^s^	p.Gln1798*	Bharadwaj *et al*.^63^
		*Het*	*MYO7A*	Missense	c.5516T > C^s^	p.Leu1839Pro	Aparisi *et al*.^8^
RP1969	USH I	***Hom***	***MYO7A***	**Frameshift**	**c.5561dupT**	p.Gln1855Alafs*56	**Novel UV4**
RP1973	USH II	***Het***	***CEP250***	**Nonsense**	**c.4006C > T** ^**s**^	p.Arg1336*	**Novel UV4**
		***Het***	***CEP250***	**Nonsense**	**c.3337A > T** ^**s**^	p.Lys1113*	**Novel UV4**
RP1979	USH II	*Het*	*USH2A*	Missense	c.10712C > T	p.Thr3571Met	Aller *et al*.^54^
		*Het*	*USH2A*	Nonsense	c.9424G > T	p.Gly3142*	Baux *et al*.^72^
RP2005	USH II	***Het***	***USH2A***	**Frameshift**	**c.4961delG**	p.Ser1654Ilefs*11	**Novel UV4**
		*Het*	*USH2A*	Nonsense	c.13822C > T	p.Arg4608*	Dreyer *et al*.^73^
RP2007	USH I	*Hom*	*PCDH15*	Nonsense	c.1737C > G^s^	p.Tyr579*	Jaijo *et al*.^58^
RP2010	USH II	*Hom*	*USH2A*	Frameshift	c.2299delG	p.Glu767Serfs*21	Liu *et al*.^59^
RP2011	USH II	*Het*	*CDH23*	Splice-site	c.6050 − 9G > A	—	von Brederlow *et al*.^74^
		***Hom***	***ADGRV1***	**CNV**	**Del. exons 28–33**	**—**	**Novel UV4**
RP2019^t^	USH	***Het***	***CDH23***	**Frameshift**	**c.8462dupT**	p.Asp2822Argfs*5	**Novel UV4**
		*Het*	*CDH23*	Missense	c.4488G > C	p.Gln1496His	Bolz *et al*.^65^
RP2022	USH II	*Het*	*USH2A*	Frameshift	c.2135delC	p.Ser712*	Bernal *et al*.^75^
		***Het***	***USH2A***	**Nonsense**	**c.6967C > T**	p.Arg2323*	**Novel UV4**
RP2023	USH II	***Het***	***USH2A***	**Nonsense**	**c.6157C > T**	p.Gln2053*	**Novel UV4**
		*Het*	*USH2A*	Frameshift	c.2299delG	p.Glu767Serfs*21	Liu *et al*.^59^
RP2028	USH	*Het*	*USH2A*	Nonsense	c.11864G > A	p. Trp3955*	Van Wijk *et al*.^76^
		***Het***	***USH2A***	**Frameshift**	**c.13778_13779insTT**	p.Val4596*	**Novel UV4**
RP2032	USH	*Het*	*USH2A*	Missense	c.2276G > T	p.Cys759Phe	Dreyer *et al*.^60^
		*Het*	*USH2A*	Missense	c.9799T > C	p.Cys3267Arg	Aller *et al*.^54^
RP2035	USH	*Hom*	*USH2A*	Frameshift	c.2299delG	p.Glu767Serfs*21	Liu *et al*.^59^
RP2037	USH	*Het*	*USH2A*	Frameshift	c.2299delG	p.Glu767Serfs*21	Liu *et al*.^59^
		*Het*	*USH2A*	Splice-site	c.949C > A	—	Pennings *et al*.^77^
RP2050	USH II	*Het*	*USH2A*	Pseudo-exon	c.7595 − 2144A > G	p.Lys2532Thrfs*56	Vaché *et al*.^17^
		*Het*	*USH2A*	Missense	c.8254G > A	p.Gly2752Arg	Nakanishi *et al*.^78^
RP2058	USH	*Het*	*USH2A*	Frameshift	c.2299delG	p.Glu767Serfs*21	Liu *et al*.^59^
		*Het*	*USH2A*	Missense	c.802G > A	p.Gly268Arg	Baux *et al*.^70^
RP2069	USH	***Het***	***MYO7A***	**IF deletion**	**c.5887_5889delTTT**	**p.Phe1963del**	**Novel UV4**
		*Het*	*MYO7A*	Missense	c.5648G > A	p.Arg1883Gln	Ouyang *et al*.^79^
RP2068	USH	***Het***	***USH2A***	**Frameshift**	**c.13102dupA**	p.Ser4368Lysfs*21	**Novel UV4**
		***Het***	***USH2A***	**Frameshift**	**c.13926dupA**	p.Gln4643Thrfs*40	**Novel UV4**
RP1936	USH	***Het***	***ADGRV1***	**Frameshift**	**c.1892delC**	p.Pro631Leufs*62	**Novel UV4**
		***Het***	***ADGRV1***	**CNV**	**Del. Exon 85**	**—**	**Novel UV4**
**Patients with only one pathogenic mutation**							
RP681	USH	***Het***	***CDH23***	**Frameshift**	**c.7279delA**	p.Thr2427Leufs*47	**Novel UV4**
RP1222	USH II	***Het***	***USH1C***	**Missense**	**c.1234G > A**	**p.Asp412Asn**	**Novel UV3**
RP1488	USH II	*Het*	*ADGRV1*	Missense	c.3151G > T	p.Asp1051Tyr	Neveling *et al*.^69^
RP1571	USH	***Het***	***CIB2***	**Missense**	**c.311G > A**	**p.Arg104Gln**	**Novel UV3**
RP2020	USH I	*Het*	*MYO7A*	Missense	c.3508G > A	p.Glu1170Lys	Cuevas *et al*.^80^
RP2034	USH	*Het*	*USH2A*	Frameshift	c.2299delG	p.Glu767Serfs*21	Liu *et al*.^59^

Het, Heterozygosis; Hom, Homozygosis; PE, Pseudoexon 40; IF, In-Frame; Dup., Duplication; Del., Deletion.

^s^Cases where segregation analysis was performed; ^t^Patients previously included in the test group.

Novel variants displayed in bold.

The novel variants found were categorized based on the guidelines of the clinical and molecular genetics society (www.emqn.org/emqn/Best+Practice) and the Unknown Variants classification system (https://grenada.lumc.nl/LOVD2/Usher_montpellier/) as pathogenic, probably pathogenic (UV4), possibly pathogenic (UV3), possibly non-pathogenic (UV2) and neutral (UV1) according to bioinformatic predictions and segregation analysis.

### Cohort of previously unscreened USH patients

We identified both pathogenic variants in 45 out of the 58 the analyzed cases and in 6 patients only one mutation was detected. No likely pathogenic mutations were found in other 7 probands.

**Figure 1:**
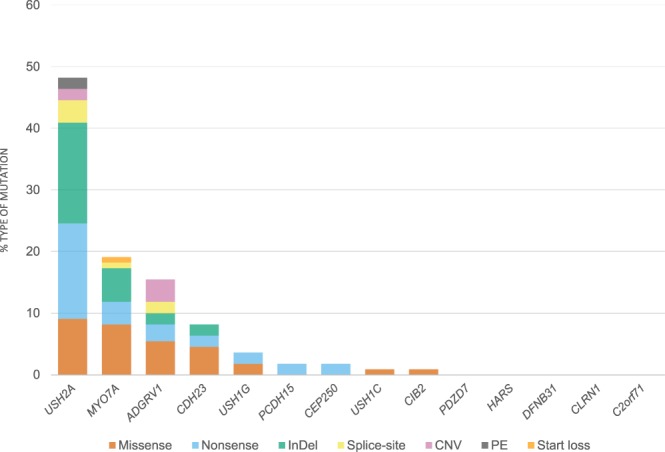
Recurrence of mutated genes included in the design of this study and distribution of the type of mutations. The data includes all the disease causative variants from the previously unscreened cohort and from the seven ultimately solved patients of the test group, which at the beginning of the study had only one causing mutation identified and the second was finally detected with the technology used in this work. Abbreviations: PE, Pseudo-exon; InDel, Insertion/Deletion; CNV, Copy Number Variation.

This work allowed to detect mutations of different nature, from which 42 were novel variants (Table 3). Among these novel changes, we were able to detect 8 missense, 14 nonsense, 11 frameshift, 4 splice-site, 1 start loss variant and 4 large rearrangements (Tables 3 and 4). Regarding all the detected mutations, *USH2A* shows the highest prevalence, accounting for 48% of the causative variants (Fig. 1).

**Table 4:** Summary of the bioinformatics predictions for the novel causative putative mutations detected in this study.

Patient	*Gene*	Nucleotide	Class	Protein function prediction tools			TIS prediction tools			Splicing impact prediction tools		
				SIFT	PPH	PROVEAN	ATGpr	NetStart	TIS Miner	HSF	MaxEnt	NNSplice
RP1350	*ADGRV1*	c.4102A > T	UV4	D	D	D	—	—	—	N	N	N
RP1353	*ADGRV1*	c.5314 − 5T > A	UV4	—	—	—	—	—	—	N	WT AS broken	Main AS not recognized
RP1540	*MYO7A*	c.1816C > T	UV4	D	D	D	—	—	—	N	N	N
RP1564	*ADGRV1*	c.14159C > T	UV4	D	D	D	—	—	—	N	N	N
RP1567	MYO7A	c.3G > A	UV4	D	N	N	TIS lost	TIS lost	TIS lost	N	N	N
RP1694	*USH1G*	c.1196T > C	UV3	D	D	N	—	—	—	N	N	N
RP1746	*USH2A*	c.12295 − 1G > A	UV4	—	—	—	—	—	—	WT AS broken	WT AS broken	Main AS not recognized
RP1857	*CDH23*	c.3115G > A	UV3	D	D	N	New TIS	New TIS	New TIS	N	N	Main DS not recognized
RP1857	*CDH23*	c.3007T > C	UV4	D	D	D	—	—	—	N	N	N
RP1869	*USH2A*	c.4385C > T	UV4	D	D	D	—	—	—	N	N	N
RP1872	*USH2A*	c.5776 + 1G > A	UV4	—	—	—	—	—	—	WT DS broken	WT DS broken	Main DS not recognized
RP1900	*MYO7A*	c.1691 − 1G > A	UV4	—	—	—	—	—	—	WT AS broken and NS created	WT AS broken	Main AS not recognized
RP1571	*CIB2*	c.311G > A	UV3	N	D	D	—	—	—	N	N	N
RP1222	*USH1C*	c.1234G > A	UV3	D	P	N	—	—	—	N	N	N

PPH, PolyPhen-2; TIS, Translation Initiation Site; D, Damaging/Probably damaging/Deleterious (SIFT/PPH/PROVEAN); P, Possibly damaging (PPH); N, Tolerated/Benign/Neutral (SIFT/PPH/PROVEAN); WT, Wild type; AS, Acceptor Site; DS, Donor Site; HSF, Human Splicing Finder.

Those variants with concurring results referred as damaging by all of the effect prediction tools of a same category were classified as UV4. The mutations were stated as UV3 when pathogenicity was assessed by two out of the three predictors. Those with neutral or UV2 prognosticated effect were not taken into account as positive results and therefore data for those cases is not shown.

Custom aCGH unveiled three CNVs in the *ADGRV1* gene: one large heterozygous duplication involving exons 79–83 in patient RP580, the heterozygous deletion of exon 85 in patient RP1936, and one large homozygous deletion comprising exons 28–33 in proband RP2011. The latter was also suspected beforehand by the null coverage of that region on the HTS sequencing results.

### Minigene splice assay analysis

**Figure 2:**
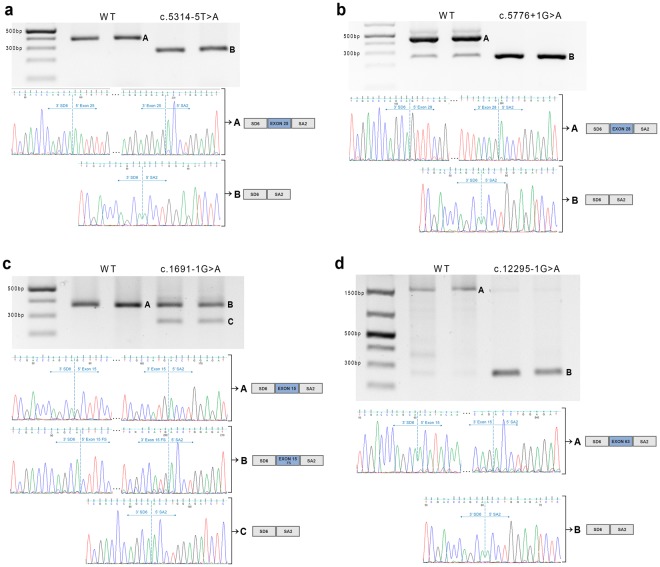
Minigene assay results for the four splicing mutations. The gel electrophoresis displays the splicing outcome of the minigene transcription for the WT and mutant alleles. *In vitro* experiments were performed in duplicate and therefore the results show both repetitions. Sanger sequencing of the results confirm the splicing processes by evidencing the transcript joints. SD6 and SA2 are the exons included in the pSPL3 exon trapping vector used in the assay. (**a**) c.5314 − 5T > A (*ADGRV1*). Band A corresponds to the correct transcript of exon 25. Band B from the mutant construction denotes the skipping of the same exon. If the transcript harboring the mutation were translated, the newly generated protein would of 1,772 aminoacids in length, p.Asn1772*. (**b**) c.5776 + 1G > A (*USH2A*). Band A is the correct transcript corresponding to the exon 28 and Band B is the skipping of the same exon. If the aberrant transcript were translated, it would generate a new truncated protein of 5,134 aminoacids in length, p.Gly1858_Thr1925del. (**c**) c.1691 − 1G > A (*MYO7A*). Band A corresponds to the correct transcript of exon 25. Band B is the aberrant splicing process due to the new site generated by the lack of a guanine at the acceptor site, entailing therefore a frameshift effect. The fragment C corresponds to de exon skipping of exon 15. If the transcript with the mutation were translated, it would generate the two proteins p. Gly564Alafs*58 and p.Phe565Argfs*11. (**d**) c.12295 − 1G > A (*USH2A*). Band A corresponds to the correct transcript of exon 63 and band B, from the mutant allele, evidences the skipping of the exon. The displayed images of the gels have been cropped to improve the clarity of the presentation, and the full-length gels are presented in Supplementary Fig. S2.

We detected 3 canonical splice-site mutations (c.1691 − 1G > A, *MYO7A*; c.5776 + 1G > A, *USH2A*; c.12295 − 1G > A, *USH2A*) and one variant with dubious consequences (c.5314 − 5T > A, *ADGRV1*) (Table 4). These variant candidates were tested through minigenes and for all of them the exon skipping was proved, confirming therefore the pathogenicity of the mutations (Fig. 2).

For the c.1691 − 1G > A *MYO7A* mutation, the minigene assay was of particular interest. Besides the skipping of exon 15, the mutant allele displayed an additional aberrant band (Fig. 2c). This additional fragment corresponds to the recognition of the first guanine of the exon as the acceptor site (the mutation is a transition of G > A), resulting in a frameshift effect starting at the first base of the exon. The *in silico* algorithms had predicted the same consequence (Table 4).

### Clinical description of RP1973

**Figure 3:**
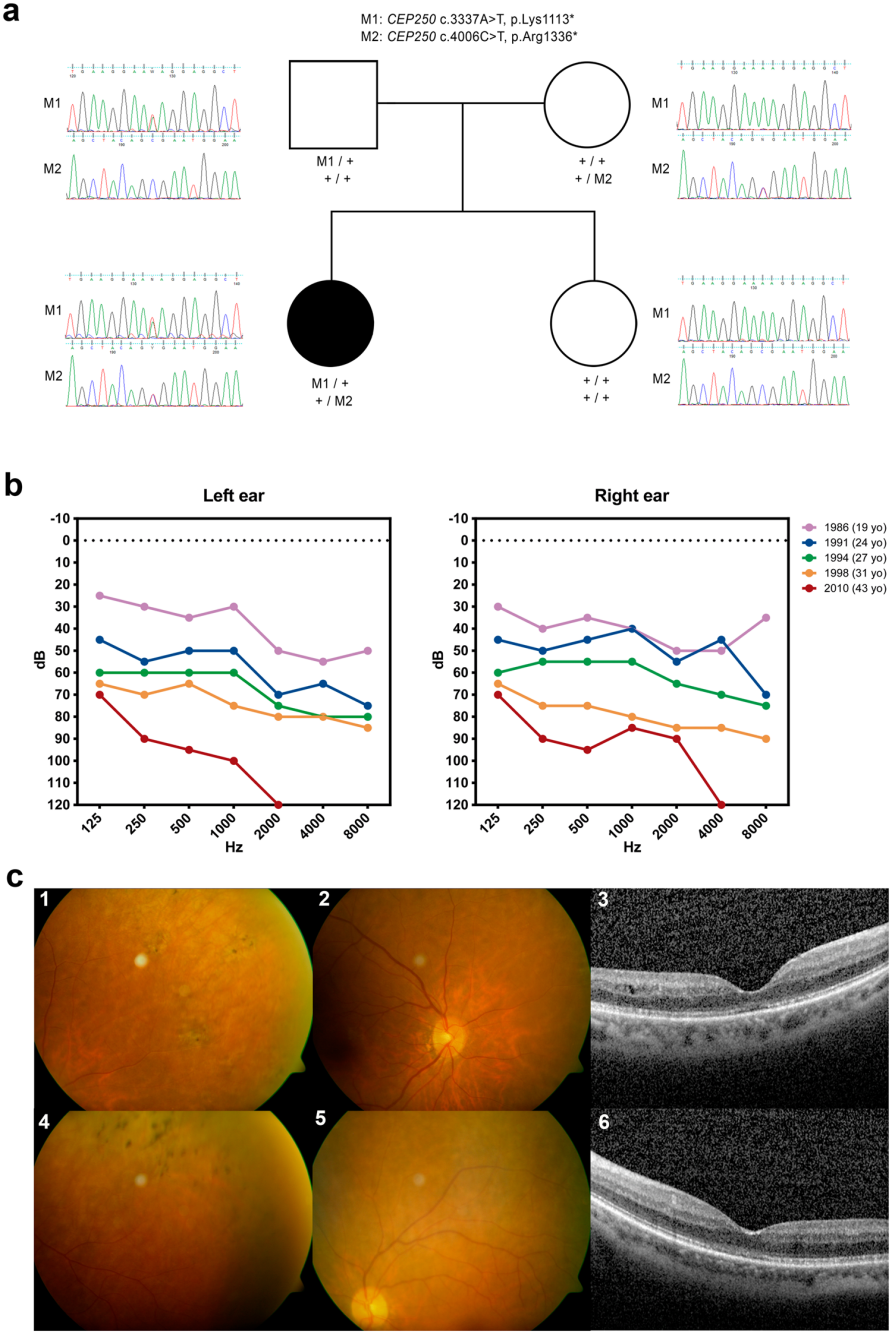
Clinical and molecular data of patient RP1973 harboring the nonsense mutations in *CEP250*. (**a**) Family pedigree with the Sanger sequencing results revealing the segregation pattern of the mutations. (**b**) Audiometric results evincing the progression of the bilateral hearing loss. (**c**) Ocular phenotype. Upper images correspond to the right eye, bottom images are from the left eye. Fundus pictures showing pigment clumps (c1, c4) and thinning of the peripheral arterioles (c2, c5). OCT images of the foveal region showing loss and discontinuity of the retinal pigment epithelium layer (c3, c6). Abbreviations: yo, years old; dB, decibel; Hz, hertz.

A remarkable case was RP1973, which was found to be a compound heterozygous for two nonsense mutations in *CEP250*. Both nonsense mutations segregate with the family, which is composed of both parents and an unaffected sibling (Fig. 3a). Patient RP1973 suffered from bilateral moderate-severe progressive hearing loss manifested at 13 years old (Fig. 3b) and late-onset progressive diminution of vision in both eyes with photophobia (first ophthalmologic examination at 44 years old). There is no history of any similar condition in any other family member. The BCVA was 0.6 in the right eye and 0.5 in the left eye (Snellen). The anterior segment findings were within normal limits, but fundus examination revealed migration of pigment in a bone-spicule pattern within a mid-peripheral annular zone of both eyes and narrowing of the peripheral retinal blood vessels (Fig. 3c). The left eye showed a glistening reflex of the inner retinal surface secondary to an epiretinal membrane. The macula of the right eye was relatively normal. Humphrey perimetry revealed peripheral visual field constriction with relative defects in the paracentral region in both eyes that has remained stable for the last five years. Macular autofluorescence images were normal in both eyes. The OCT shows a normal macular thickness with discontinuity of the outer segment layer of the photoreceptors around the foveal center in both eyes (Fig. 3c). Full-field electroretinography showed only mild alterations in the scotopic flash electroretinography (ERG), as the amplitudes of the b-wave were reduced in the right eye and absent in the left eye. Macular ERG showed an absence of response in both eyes, and Visual Evoked Potentials (VEP) were altered (Supplemental Fig. S1). Due to the symptoms, the nature of the variants leading to a premature stop codon and the co-segregation analysis we consider both mutations as disease causing for an USH-like phenotype.

The analysis of the targeted panel and WES results showed no additional putative pathogenic mutations, except for one heterozygous missense variant in *USH2A* (rs773526991: c.4561C > T/p.Arg1521Cys), presenting an allele frequency of 0.00002 in ExAC and for which the *in silico* tools implied a deleterious effect. Nevertheless, no other potential mutation was identified in *USH2A*.

## Discussion

Usher syndrome is genetically heterogeneous mostly due to the high number of genes involved and their large size. The genetic etiopathogeny relies on all kinds of mutations among these genes and additionally, most of the variants are private. For that reason, it is very difficult to perform molecular diagnosis by conventional genotyping or direct gene sequencing. In our study, we were able to detect biallelic mutations in an USH gene in 45 out of the 58 previously unscreened patients (77.6%) and we identified 96 out of the 116 expected mutated alleles (82.8% detection ratio). That percentage difference is due to the fact that 6 cases were carriers of only one pathogenic variant (Table 3). The remaining undiscovered second mutation, as well as both variants of unresolved cases, may be located either in other USH responsible still unknown genes or in other non-coding regions that were not incorporated in our design. The pathogenic deep intronic mutation c.7595 − 2144A > G in *USH2A* was included in the study, but five other have been recently designated to be pathogenic^27–29^, which proves that still many deep intronic mutations may remain unveiled and further whole gene screen studies are of great interest.

The output of the analysis of the raw data is very dependent on the algorithm used for the mapping and variant calling. Two control variants, consisting of frameshift duplications, were detected only when relaxing the software quality parameters, suggesting a possible hindrance for the Ion Torrent mapping algorithm to align homopolymers. Indeed, other studies have reported these homopolymer-associated errors and even over and under-calling errors in non-homopolymer regions^32,33^. Additional factors for this technology suggested by other authors, such as the biases produced by the GC content or the underestimation of the quality scores^32,34^, probably contribute to the false negative calling errors.

The platform and panel design provided a 91% reliability based on the point mutation detection rate of the test group, but it reached a 100% of accuracy when thresholds of the mapping and annotation variables were decreased. However, no additional causative variants were found in the group of unresolved cases after applying the same procedure. Nevertheless, the failure to detect these variants could also fall on the HTS system used for the study, escaping variant detection independently on the resulting data management.

Among the study, two patients from the previously unscreened group presented mutations in genes that were not consistent with their clinical diagnosis, being these genes usually responsible for another USH subtype. One USH I patient (RP1748) carried biallelic mutations in *USH2A* and an USH II case in the *MYO7A* gene (RP1567). Still, the event of a molecular diagnosis not quite matching the clinical phenotype is not unusual and has been previously reported in other studies^8,35^. Indeed, this supports the further investigation of USH patients by HTS to establish better genotype – phenotype associations.

Many previous studies have evidenced the presence of large rearrangements among different USH populations, establishing them as a significant genetic alteration causing the disease. *PCDH15* and *USH2A* are the most common genes displaying such CNVs^30,36^, but also large rearrangements have been found in *MYO7A*, *CDH23* and *ADGRV1*^37,38^.

A CNVs survey based on the coverage of the sequencing results was not possible due to the wide deviation of the target enrichment technique by *loci* amplification. However, large homozygous deletions could be inferred from null covered regions corresponding to several adjacent probes, when observed in punctual cases. The supplemental analysis by MLPA or aCGH allowed us to detect a total of five large rearrangements among the test group and the previously unscreened cohort, four of these rearrangements being novel. Concerning our series of patients without prior genetic diagnosis, the CNVs account for 5.2% of the total identified pathogenic alleles.

Four of the novel mutations were intronic variants located in splicing regions that, though all but one were set on canonical ±1 *loci*, a sort of functional analysis would provide further support of their pathogenicity. Certainly, the minigene assays proved that all these four mutations cause an aberrant splicing.

Regarding the compound heterozygous case for the two nonsense mutations in *CEP250*, our study provides sufficient data for the gene to be classified as USH-like causative. The association was firstly introduced in a study of a consanguineous family of Iranian Jewish origin characterized by early onset hearing loss and mild RP^7^ and, very recently, Kubota *et al*.^39^ presented a Japanese family carrying compound heterozygous nonsense mutations in *CEP250*, with a clinical phenotype of cone-rod dystrophy and sensorineural hearing loss. Our patient with the *CEP250* mutations (RP1973) presented with progressive hearing loss and mild macular affectation with lowering of the visual acuity and photophobia, which are similar symptoms to those of the latter work, thus consolidating its role as a gene responsible for mimicking Usher syndrome. It has to be remarked that RP1973 shows a clearly progressive SNHL, yet the aforementioned studies do not give any details about the deafness evolution and, thus, a full comparative analysis is not feasible. There is another study correlating *CEP250* with non-syndromic RP (nsRP) due to a detected homozygous missense mutation^40^. These findings are in agreement with ours and other authors observations that different diseases can be caused by the same gene depending on specific mutations, such as *USH2A* that can cause either nsRP or USH, or the USH genes *MYO7A*, *USH1C*, *CDH23*, *PCDH15*, *USH1G*, *WHRN* or *CIB2* that can cause non syndromic hearing loss or USH^41–48^. In view of the different but closely related phenotypes associated to *CEP250*, thorough clinical examinations of the cases should be performed to better understand the consequences of mutations in this gene, particularly those regarding cone affectation.

In the last years, the USH molecular diagnosis through HTS approaches have replaced the traditional techniques based on Sanger sequencing^35,49^. The more recent next generation sequencing approaches enable a detection ratio between 50–100%, depending on the cohort and design of study^8,9,12,50–53^. Here, we provide a HTS method based on targeted exome library generation by amplification and the subsequent ion sensing-based sequencing that allows an average allele detection ratio compared to the other mentioned studies. It is, though, unfair to compare these varying efficiencies, since they do not only rely on the sequencing system, but also on the cohort selection criteria of the samples. For instance, the group analyzed by Qu *et al*.^53^, Besnard *et al*.^50^ and Eandi *et al*.^9^ consisted of only five, thirteen and seventeen probands respectively, a rather scarce number of samples that might bias the efficiency outcome. Additionally, those and other studies such as Aparisi *et al*.^8^ and Bonnet *et al*.^51^ involved only well USH characterized patients. Our study not only included a larger number of samples, but also some unclassified USH cases. Therefore, another partial reason for the unsolved cases could be the misdiagnosis of some patients as USH, who could harbor several mutations in other genes that together may mimic the syndrome. From the seven unresolved patients without genetic diagnosis, three lack in detailed clinical notes that clearly support the cases as USH. The remaining four patients do not fully harmonize with the syndrome, since they present a late-onset hearing impairment. If we were not to take these samples into account, the detection ratio would increase from the 82.8% up to 94.1%. Even displaying such a solid outcome, this HTS approach falls short of CNV detection, yet it allows the use of only 10 ng of starting DNA (admitting as well some degradation). All these features shall be taken in consideration, depending on the requirements and resources of each center and the group of study.

Our updated custom design for USH targeted exome sequencing is a reliable tool for molecular diagnosis of the disease, and its implementation in the health care system would lead to a great profit for the patients. Furthermore, *CEP250* should be officially recognized as a gene causative of Usher-like syndrome.

## Electronic supplementary material

Supplementary Information

## Data Availability

The datasets generated and analysed during the current study are available from the corresponding author on reasonable request.
