# Supplementary material for: High-throughput sequencing for the molecular diagnosis of Usher syndrome reveals 42 novel mutations and consolidates CEP250 as Usher-like disease causative
Source: Sci Rep. 2018 Nov 20;8:17113. doi: 10.1038/s41598-018-35085-0 (PMC6244211; doi:10.1038/s41598-018-35085-0)
Supplement: Supplementary file 1 — Supplementary Information [file 41598_2018_35085_MOESM1_ESM.pdf]

High-throughput sequencing for the molecular diagnosis of Usher syndrome reveals 42 novel mutations and consolidates *CEP250* as Usher-like disease causative

Carla Fuster-García[1], Gema García-García*[1,2], Teresa Jaijo[1,2,3], Neus Fornés[1]; Carmen Ayuso[2,4], Miguel Fernández-Burriel[5], Ana Sánchez-De la Morena[6], Elena Aller[#1,2,3], José M Millán[#1,2].

[1]Grupo de Investigación en Biomedicina Molecular, Celular y Genómica, Instituto de Investigación Sanitaria La Fe (IIS La Fe), Valencia, Spain. [2]CIBER de Enfermedades Raras (CIBERER), Madrid, Spain. [3]Unidad de Genética y Diagnóstico Prenatal, Hospital Universitario y Politécnico La Fe, Valencia, Spain. [4]Servicio de Genética, Fundación Jiménez Díaz, University Hospital, Instituto de Investigación Sanitaria Fundación Jiménez Díaz IIS-FJD, UAM, Madrid, Spain. [5]Unidad de Genética, Hospital de Mérida, Mérida, Badajoz, Spain. [6]Servicio de Oftalmología, Hospital de Mérida, Mérida, Badajoz, Spain.

[#]These authors contributed equally to this work

*Corresponding Author, e-mail: gegarcia@ciberer.es

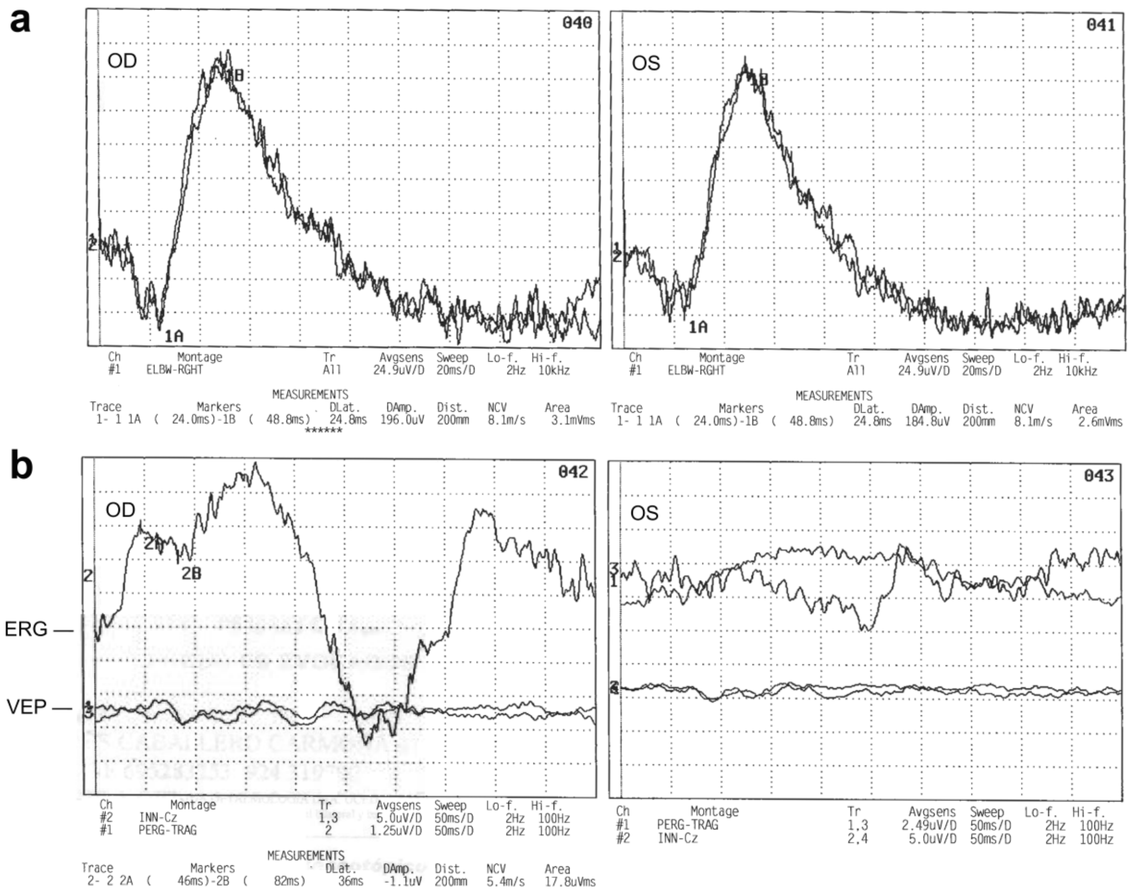

Supplemental Figure S1. ERG results.

(**a**) Only light alterations are seen in dark adapted at 3 cd (cone and rod mixed response).

(**b**) In the macular light adapted, the alterations are more severe, affecting also the VEPs.

Abbreviations: OD, right eye; OS, left eye.

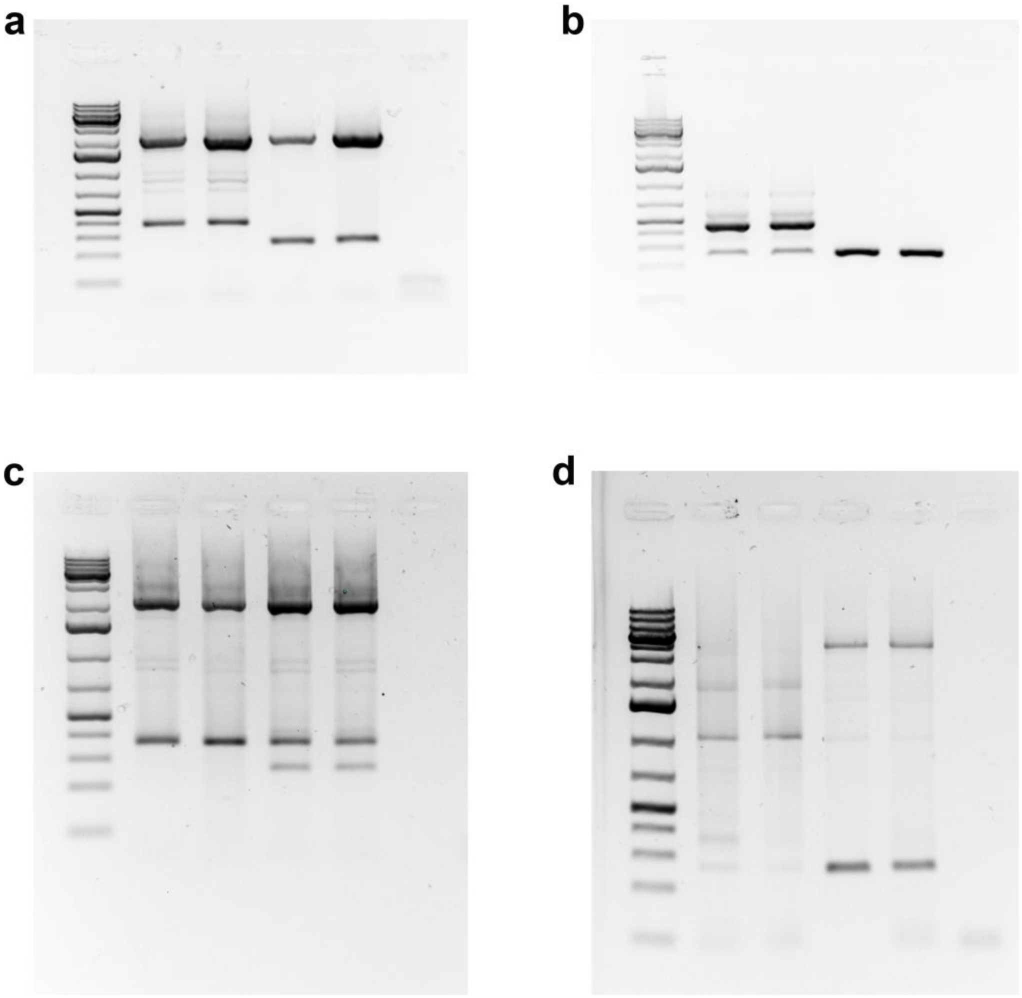

Supplemental Figure S2. Full-length gels from Figure 2.

Gel corresponding to the minigene assay for the (**a**) c.5314-5T>A mutation (*ADGRV1*), (**b**) c.5776+1G>A mutation (*USH2A*), (**c**) c.1691-1G>A mutation (*MYO7A*), (**d**) c.12295-1G>A mutation (*USH2A*)**.**
